# History of Having a Macrosomic Infant and the Risk of Diabetes: The Japan Public Health Center-Based Prospective Diabetes Study

**DOI:** 10.1371/journal.pone.0084542

**Published:** 2013-12-19

**Authors:** Yusuke Kabeya, Atsushi Goto, Masayuki Kato, Yoshihiko Takahashi, Yumi Matsushita, Manami Inoue, Tetsuya Mizoue, Shoichiro Tsugane, Takashi Kadowaki, Mitsuhiko Noda

**Affiliations:** 1 Department of Diabetes Research, Diabetes Research Center, National Center for Global Health and Medicine, Tokyo, Japan; 2 Department of Internal Medicine, Saiseikai Central Hospital, Tokyo, Japan; 3 Department of Clinical Research Coordination, Center for Clinical Sciences, National Center for Global Health and Medicine, Tokyo, Japan; 4 Division of Diabetes and Metabolism, Iwate Medical University School of Medicine, Morioka, Japan; 5 Epidemiology and Prevention Division, Research Center for Cancer Prevention and Screening, National Cancer Center, Tokyo, Japan; 6 AXA Department of Health and Human Security, Graduate School of Medicine, The University of Tokyo, Tokyo, Japan; 7 Department of Epidemiology and Prevention, Center for Clinical Sciences, National Center for Global Health and Medicine, Tokyo, Japan; 8 Department of Diabetes and Metabolic Diseases, The University of Tokyo, Tokyo, Japan;; University of Sao Paulo, Brazil

## Abstract

**Objective:**

The aim of the present study was to test a hypothesis that a history of having a macrosomic infant (≥4000g) is associated with the risk of diabetes.

**Methods:**

Data on the Japan Public Health Center-based Prospective diabetes cohort were analyzed, which is a population-based cohort study on diabetes. The survey of diabetes was performed at baseline and at the 5-year follow-up. A history of having a macrosomic infant was assessed using a self-administered questionnaire. A cross-sectional analysis was performed among 12,153 women who participated in the 5-year survey of the cohort. Logistic regression was used to examine the relationship between a history of having a macrosomic infant and the presence of diabetes. A longitudinal analysis was also conducted among 7,300 women without diabetes who participated in the baseline survey. Logistic regression was used to investigate the relationship between a history of having a macrosomic infant and the incidence of diabetes between the baseline survey and the 5-year survey.

**Results:**

In the cross-sectional analysis, parous women with a positive history were more likely to have diabetes in relation to parous women without (OR = 1.44, 95% CI = 1.13-1.83). The longitudinal analysis showed a modest but non-significant increased risk of developing diabetes among women with a positive history (OR = 1.24, 95% CI = 0.80-1.94).

**Conclusions:**

An increased risk of diabetes was implied among women with a history of having a macrosomic infant although the longitudinal analysis showed a non-significant increased risk.

## Introduction

Changes in metabolic conditions occur in women during pregnancy, which enables them to supply nutrients preferentially to fetus. One of these changes arises in glucose metabolism. Pregnancy is commonly recognized as a state of physiological and temporary insulin resistance. Under such conditions, some women who have poor β-cell compensation might experience an increase in plasma glucose levels.

Maternal hyperglycemia, which leads to an elevated placental glucose transfer to the fetus, could induce fetal overgrowth. Past studies [1–3] have reported that not only maternal diabetes but also slight glucose intolerance are a risk factor for developing fetal overgrowth. One study [4] reported that there was a positive and continuous relationship between maternal glycemia and size at birth weight. Therefore, it is expected that women who had a macrosomic infant were more likely to have had elevated plasma glucose levels during pregnancy than those without. These women might have an underlying β-cell dysfunction and be likely to develop diabetes later in life. In other words, a medical history of having a macrosomic infant could be a risk factor of developing diabetes. In fact, a population-based study in the US [5] reported the usefulness of a simple questionnaire including a history of having a macrosomic infant to identify individuals at high-risk for undiagnosed diabetes. However, there have been few studies which reported the risk estimates for the relationship between the medical history and the risk of developing diabetes. 

In the present study, we tested a hypothesis that a history of having a macrosomic infant (≥4000g) is associated with the risk of diabetes and calculated the risk estimates for the relationship. For this purpose, the Japan Public Health Center-based Prospective (JPHC) diabetes cohort, which consists of registered inhabitants in public health center (PHC) areas across Japan, was examined at baseline (1998-2000) and a 5-year follow-up (2003-2005), using a standardized questionnaire including a reproductive history and laboratory measurements.

## Materials and Methods

### General Scheme

 In the present study, we performed both a cross-sectional analysis and a longitudinal analysis to examine the relationship. First, we cross-sectionally analyzed the relationship between a history of having a macrosomic infant and the presence of diabetes among the women who participated in the 5-year survey. Since information on history of childbirth was incomplete in the questionnaire administered at baseline, the cross-sectional analysis was performed in the 5-year survey. However, the cross-sectional design had a methodological issue regarding reverse causality. There could be a possibility that a woman who have had established diabetes before pregnancy might have delivered a macrosomic infant. To address this issue, we focused on women without diabetes in the baseline survey and longitudinally observed the incidence of diabetes between the baseline survey and 5-year survey. Then, the relationship between a history of having a macrosomic infant and the incidence of diabetes was analyzed.

### Study population

Data from the JPHC Study, which was a large longitudinal cohort study in Japan investigating cancer, cardiovascular disease and other lifestyle-related diseases, were used in the present study. The details of the study design have been described elsewhere [6]. In brief, the JPHC Study was initiated in 1990 for Cohort I, and added in 1993 for Cohort II. The study population consists of all registered Japanese inhabitants in 11 PHC areas aged 40-59 years old in Cohort I and 40-69 years old in Cohort II at the start of each survey. The baseline survey of the diabetes study (the JPHC Diabetes Study) was performed in 1998-1999 for Cohort II and in 2000 for Cohort I. Participants received the annual health checkups in each PHC-administered area, and a self-administered questionnaire specific to diabetes research and measurement of HbA1c were added to their routine health checkup examination. The 5-year follow-up survey for Cohort II was conducted in 2003-2004 and for Cohort I in 2005, respectively. 

A total of 18,049 women participated in the baseline survey and 12,597 in the 5-year survey. Then, 8,046 women participated in the both surveys. The participants provided their written informed consent to participate in this study. This study was approved by an ethics committee of the International Medical Center of Japan, which was a former name of National Center for Global Health and Medicine.

#### 1: Subjects included in the cross-sectional analysis

**Figure 1:**
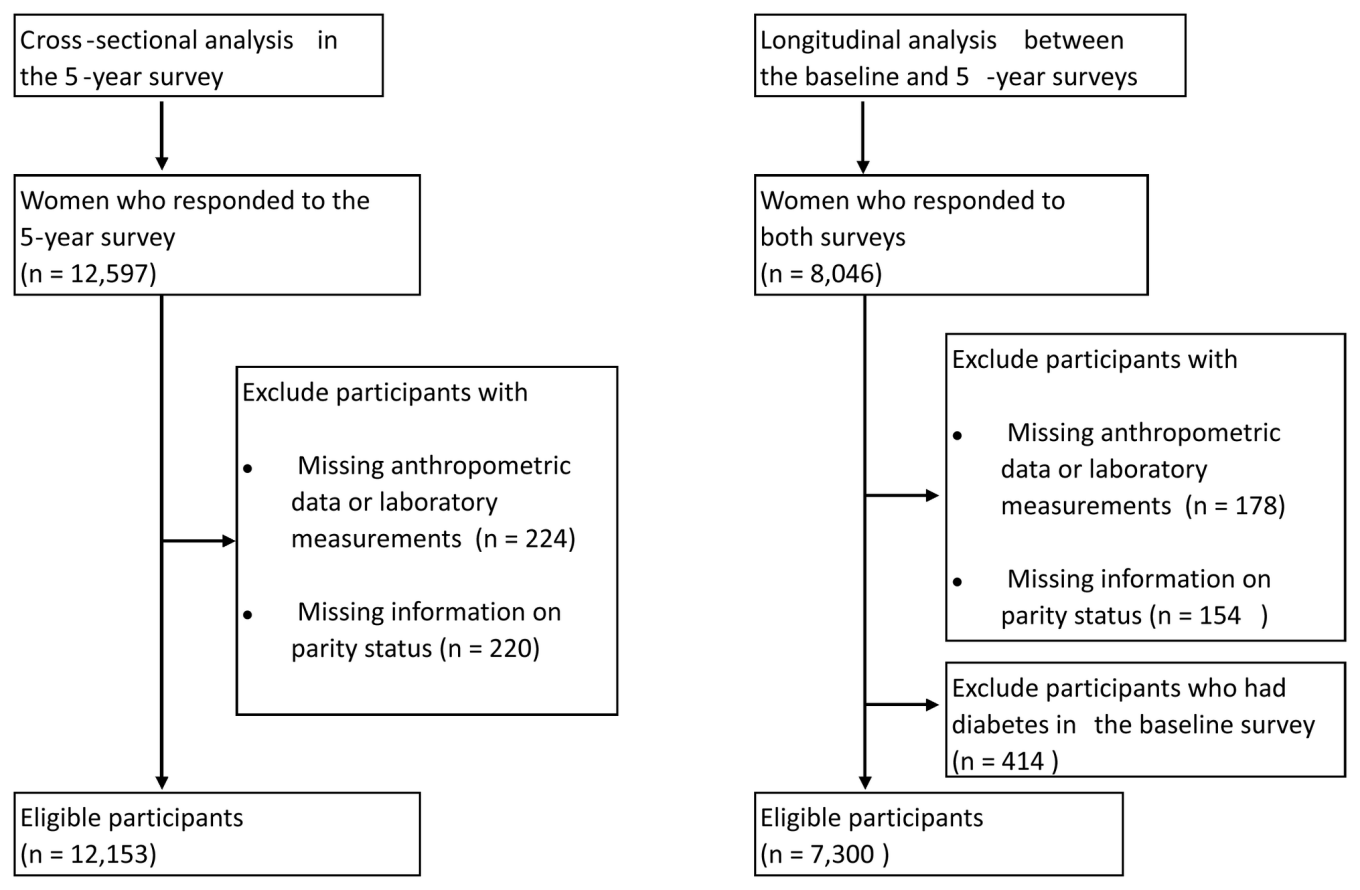
Study flow chart.

 Of the 12,597 women who participated in the 5-year survey, 224 were excluded because of missing data on anthropometric or laboratory measurements and 220 were excluded because of missing data on parity status. Then, a total of 12,153 women were included in the cross-sectional analysis (Figure 1). 

#### 2: Subjects included in the longitudinal analysis

The analysis was restricted to those who participated in the both baseline and 5-year surveys. Of the 8,046women, 178 were excluded because of missing data on anthropometric or laboratory measurements and 154 were excluded because of missing data on parity status. Furthermore, 414 women who had diabetes at the time of the baseline survey were excluded. Then, a total of 7,300 women without diabetes were included in the longitudinal analysis (Figure 1). 

### History of childbirth

As mentioned previously, since information on history of childbirth was incomplete in the questionnaire administered at baseline, the information was obtained from the questionnaire in the 5-year survey. Subjects were classified into three categories according to parity status: nulliparous women, parous women with a history of having a macrosomic infant, and parous women without.

### Case ascertainment of diabetes

In the present study, diabetes was diagnosed when a participant met any of the following criteria: 1) self-reported diabetes, 2) a fasting plasma glucose (FPG) value of 126 mg/dL or more, and 3) a casual plasma glucose value of 200 mg/dL or more, 4) an HbA1c value of 6.5 % or more. A fasting blood sample, which was defined as a sample collected ≥ 8 hours after the last caloric intake, was collected from 4,317 women in the cross-sectional analysis and 1,175 women in the baseline of the longitudinal analysis. Otherwise a blood sample was collected as a casual blood sample.

### Other characteristics of the study participants

 Body mass index (BMI), family history of diabetes and physical activity were obtained from the questionnaire in the baseline and 5-year surveys. The validity of self-reported BMI in the JPHC study has been examined and published previously [7]. The self-reported BMIs were slightly lower than the measured BMIs and the Spearman correlation coefficients were 0.89 in men and 0.91 in women. Family history of diabetes was defined as the presence of at least one relative with diabetes. Physical activity was assessed by self-reported daily walking time. Daily walking time was categorized into five (<0.5, 0.5-0.9, 1-1.9, 2-2.9 and 3hr or more) in the cross-sectional analysis and four (<0.5, 0.5-0.9, 1-1.9, and 2hr or more) in the baseline of the longitudinal analysis. The categorizations were based on the questionnaires used in the baseline and 5-year surveys.

### Statistical analysis

We performed two analyses to investigate the relationship: 1) a cross-sectional analysis to examine the relationship between a history of having a macrosomic infant ( ≥4000g ) and the presence of diabetes in the 5-year survey, 2) a longitudinal analysis to examine the relationship between a history of having a macrosomic infant and the incidence of diabetes between the baseline survey and 5-year survey. In each analysis, characteristics of the subjects were categorized according to parity status (nulliparous women, parous women with a history of having a macrosomic infant, and parous women without). As for the comparison of variables between the three groups, one-way analysis of variance (ANOVA) tests and pairwise t-tests were used for comparing continuous variables while χ^2^ tests were used for comparing categorical variables. Pairwise comparisons were performed using the Bonferroni method to investigate which group difference was statistically significant. Since triglycerides did not follow the Normal distribution, log-transformed values were used for the comparison.

In the cross-sectional analysis, logistic regression was performed to calculate the odds of having diabetes among women with a positive history in relation to those without. The analysis was adjusted for age, BMI, systolic blood pressure (BP), family history of diabetes, and daily walking time.

In the longitudinal analysis, the analysis was restricted to those who participated in both the baseline and 5-year surveys. The age-adjusted cumulative incidences of diabetes between the baseline survey and the 5-year survey were calculated according to parity status. The age-standardization was conducted using the direct method. They were standardized to Japanese model population in 1985. Then, a logistic regression analysis was performed to calculate the odds of developing diabetes among women with a positive history in relation to those without, which was adjusted for age, BMI, systolic BP, family history of diabetes, and daily walking time recorded in the baseline survey. All analyses were performed using Stata version 11 for Windows (Stata Corp., Texas, USA).

## Results

### Descriptive epidemiology of a history of having a macrosomic infant

Of the 12,153 women who participated in the cross-sectional analysis, 847 women (7.0%) were nulliparous and 725 women (6.0 %) had a history of having a macrosomic infant. The remaining 10,581 women were parous but did not have a positive history. 

As for the longitudinal analysis, of the 7,300 women without diabetes at baseline, 489 (6.7%) women were nulliparous and 405 (5.5%) women had a history of having a macrosomic infant. The remaining 6,406 women were parous but did not have a positive history.

### Characteristics of subjects in the cross-sectional analysis

Characteristics of the subjects in the cross-sectional analysis are shown in Table 1. In the analysis, age, BMI, systolic BP, the proportion of diabetes, the proportion of subjects with a positive family history of diabetes and daily walking time were significantly different between the three groups. The prevalence of diabetes in parous women with a history of having a macrosomic infant was 12.6%, while the prevalence was 8.9 % in parous women without and 9.0% in nulliparous women.

**Table 1:** Characteristics of women according to parity status in the 5-year cross-sectional analysis.

Characteristics	Overall	Nulliparous women	Parous women			Comparison			
			History of having a macrosomic infant			Between three groups	Pairwise		
			No		Yes	*P-value**^1^***	*P*-value**^*2*^**	*P*-value**^*2*^**	*P*-value**^*2*^**
		(Group 1)	(Group 2)		(Group 3)		*1 vs 2*	*2 vs 3*	*1 vs 3*
Number of subjects	12,153	847	10,581		725				
Age (SD)	66.0 (6.9)	66.2 (7.0)	66.0 (6.9)		65.3 (6.7)	0.010	1.000	0.010	0.031
BMI (SD)	23.9 (3.4)	23.7 (3.5)	23.9 (3.4)		24.6 (3.4)	<0.001	0.458	<0.001	<0.001
Systolic BP (SD), mmHg	130 (17)	130 (17)	130 (17)		128 (16)	0.001	1.000	0.015	0.001
Diastolic BP (SD), mmHg	76 (10)	76 (10)	76 (10)		76 (10)	0.761			
HbA1c (SD), %	5.75 (0.68)	5.72 (0.60)	5.75 (0.68)		5.79 (0.69)	0.104			
Casual PG, mg/dl	107.9 (27.7)	106.5 (26.7)	108.0 (27.7)		108.6 (28.4)	0.254			
Fasting PG (SD), mg/dl (n = 4,317)	100.5 (20.1)	99.3 (17.2)	100.4 (20.1)		103.1 (23.3)	0.060			
Diabetes, n (%)	1,109 (9.1)	76 (9.0)	942 (8.9)		91 (12.6)	0.004	1.000	0.003	0.066
FH of diabetes, n (%)	2,266 (18.7)	177 (20.9)	1,935 (18.3)		154 (21.2)	0.031	0.141	0.180	0.868
Daily walking time, n (%)									
<0.5 hrs	2,529 (20.8)	201 (23.7)	2,171 (20.5)		157 (21.7)	<0.001	<0.001	0.222	<0.001
0.5-0.9 hrs	2,647 (21.8)	213 (25.2)	2,296 (21.7)		138 (19.0)				
1.0-1.9 hrs	1,850 (15.2)	157 (18.5)	1,600 (15.1)		93 (12.8)				
2.0-2.9 hrs	1,348 (11.1)	109 (12.9)	1,161 (11.0)		78 (10.8)				
≥3.0 hrs	3,779 (31.1)	167 (19.7)	3,353 (31.7)		259 (35.7)				

^1^ The three groups were compared using one-way analysis of variance for continuous variables and χ^2^tests for categorical variables.

^2^ Pairwise comparisons were performed using the Bonferroni method.

BP: blood pressure, FH: family history, PG: plasma glucose

Mean HbA1c in parous women with a positive history was 5.79% and that in parous women without was 5.75%. The adjusted difference in mean HbA1c between them was not statistically significant (Table 2).

**Table 2:** Mean HbA1c levels among subjects with different parity status in the 5-year cross-sectional analysis.

	Mean HbA1c (%)	Difference in HbA1c (%)	95%CI	Adjusted* difference (%)	95%CI
Total	5.75 (0.68)				
Parous women **without** a history of having a macrosomic infant	5.75 (0.68)	reference		reference	
Nulliparous women	5.72 (0.60)	-0.03	(-0.08 to 0.02)	-0.05	(-0.10 to -0.01)
Parous women **with** a history of having a macrosomic infant	5.79 (0.69)	0.04	(-0.01 to 0.09)	0.03	(-0.02 to 0.08)

^1^ Adjusted for age, BMI, systolic BP, family history of diabetes and walking time.

### History of having a macrosomic infant and the presence of diabetes in the cross-sectional analysis

Table 3 depicts the results of the logistic regression analyses in the cross-sectional analysis. Parous women with a positive history were more likely to have diabetes in relation to parous women without [odds ratio (OR) = 1.47, 95% CI = 1.17-1.85]. The OR was slightly attenuated after adjustment for age, BMI, systolic BP, family history of diabetes, and daily walking time (OR = 1.44, 95% CI = 1.13-1.83). Nulliparous women did not show a significant change in the odds of having diabetes in relation to parous women without a positive history (OR = 0.94, 95%CI = 0.73-1.22). After excluding nulliparous women, the adjusted OR among parous women with a positive history in relation to parous women without was calculated again, which did not change at all (OR = 1.44, 95% CI = 1.13-1.83).

**Table 3:** History of having a macrosomic infant and ORs of having diabetes in the 5-year cross-sectional analysis.

		Diabetes							
		No	Yes		Crude OR	95%CI		Adjusted OR**^*1*^**	95%CI
		n=11,044	n=1,109						
Parous women **without** a history of having a macrosomic infant		9,639	942		1.00			1.00	
Nulliparous women		771	76		1.01	(0.79-1.29)		0.94	(0.73-1.22)
Parous women **with** a history of having a macrosomic infant		634	91		1.47	(1.17-1.85)		1.44	(1.13-1.83)

^1^ Adjusted for age, BMI, systolic BP, family history of diabetes and daily walking time.

### History of having a macrosomic infant and the incidence of diabetes in the longitudinal analysis

Characteristics of the subjects included in the longitudinal analysis were shown in Table 4. Age, BMI, HbA1c levels and daily walking time were significantly different between the three groups. The age-adjusted cumulative incidences of diabetes during the 5 years were 6.8 % in parous women with a positive history, 4.6% in parous women without and 4.7% in nulliparous women. In the logistic regression analysis, parous women with a positive history had a modest but non-significant increase in the risk of developing diabetes with an OR of 1.27 (95% CI = 0.81-1.93) in relation to parous women without. Adjustment for age, BMI, systolic BP, family history of diabetes, and daily walking time did not bring a substantial change in the risk estimates (OR = 1.24, 95% CI = 0.80-1.94). With regard to nulliparous women, their risk of developing diabetes was almost equivalent to parous women with a positive history (OR = 0.99, 95% CI = 0.63-1.55) (Table 5). After excluding nulliparous women, the adjusted OR among parous women with a positive history in relation to parous women without was calculated, which brought little change (OR = 1.23, 95% CI = 0.80-1.92).

**Table 4:** Characteristics of women according to parity status at baseline in the longitudinal analysis.

Characteristics	Overall	Nulliparous women	Parous women			Comparison			
			History of having a macrosomic infant			Between three groups	Pairwise		
			No		Yes	*P-value**^1^***	*P*-value**^*2*^**	*P*-value**^*2*^**	*P*-value**^*2*^**
		(Group 1)	(Group 2)		(Group 3)		*1 vs 2*	*2 vs 3*	*1 vs 3*
No. of subjects	7,300	489	6,406		405				
Age (SD), years	61.6 (6.7)	61.9 (6.9)	61.7 (6.7)		60.8 (6.6)	0.033	1.000	0.046	0.047
BMI (SD), kg/m^2^	23.7 (3.1)	23.4 (3.4)	23.7 (3.1)		24.3 (3.1)	<0.001	0.192	0.001	<0.001
Systolic BP (SD), mmHg	129 (17)	130 (18)	129 (17)		128 (16)	0.135	1.000	0.196	0.186
Diastolic BP (SD), mmHg	77 (10)	76 (10)	77 (10)		77 (10)	0.870			
HbA1c (SD), %	5.44 (0.39)	5.42 (0.39)	5.44 (0.39)		5.49 (0.37)	0.016	0.828	0.030	0.019
Casual PG (SD), mg/dl	100.7 (17.1)	100.5 (17.8)	100.7 (17.1)		100.9 (16.8)	0.955			
Fasting PG (SD), mg/dl (n = 1,175)	93.7 (8.7)	93.4 (9.8)	93.7 (8.5)		94.5 (9.5)	0.709			
FH of diabetes, n (%)	1,124 (15.4)	78 (16.0)	984 (15.4)		62 (15.3)	0.934			
Daily walking time, n (%)									
<0.5 hrs	990 (13.6)	83 (17.0)	847 (13.2)		60 (14.8)	0.037	0.090	0.690	0.117
0.5-0.9 hrs	1,602 (22.0)	120 (24.5)	1,410 (22.0)		72 (17.8)				
1.0-1.9 hrs	1,629 (22.3)	96 (19.6)	1,437 (22.4)		96 (23.7)				
≥2.0 hrs	3,079 (42.2)	190 (38.9)	2,712 (42.3)		177 (43.7)				

^1^ The three groups were compared using one-way analysis of variance for continuous variables and χ^2^tests for categorical variables.

^2^ Pairwise comparisons were performed using the Bonferroni method.

BP: blood pressure, FH: family history, PG: plasma glucose

**Table 5:** History of having a macrosomic infant and incidence of diabetes in the longitudinal analysis.

	Diabetes							
	No	Yes	Incidence of diabetes during the 5 years**^*1*^**	Crude OR	95%CI		Adjusted OR**^*2*^**	95%CI
	n=6,966	n=334						
Parous women **without** a history of having a macrosomic infant	6,117	289	4.6%	1.00			1.00	
Nulliparous women	467	22	4.7%	0.99	(0.64-1.55)		0.99	(0.63-1.55)
Parous women **with** a history of having a macrosomic infant	382	23	6.8%	1.27	(0.82-1.97)		1.24	(0.80-1.94)

^1^ Standardized to Japanese model population in 1985.

^2^ Adjusted for age, BMI, systolic BP, family history of diabetes and daily walking time.

### Characteristics of women who had diabetes at baseline

 To analyze the discrepancy in the results between the cross-sectional analysis and the longitudinal analysis, the characteristics of women who were excluded from the longitudinal analysis because of the presence of diabetes at baseline (n = 414) were described. They were compared with the women who were included in the longitudinal analysis (n = 7,300). Table 6 shows the characteristics of women according to their diabetes status at baseline and at 5-year follow-up. Women who had diabetes at baseline and were excluded from the longitudinal analysis had a high percentage of having a positive history (9.2%). They were more likely to have family history of diabetes and seemed to be less active. They had higher systolic BP and worse glycemic control than women who were included in the longitudinal analysis.

**Table 6:** Characteristics of women according to their diabetes status at baseline and at 5-year follow-up in the Japan Public Health Center-based Prospective study.

	Diabetes at baseline	Yes	No	No	
	Diabetes at 5-year follow-up		Yes	No	*P* for trend
Characteristics	Inclusion in the longitudinal analysis	Excluded	Included	Included	
Number of subjects			334	6966	
Age (SD)		63.6 (6.3)	62.4 (6.7)	61.6 (6.7)	<0.001
BMI (SD)		24.7 (3.8)	25.1 (3.7)	23.6 (3.1)	<0.001
Systolic BP (SD), mmHg		134 (18)	134 (17)	129 (17)	<0.001
Diastolic BP (SD), mmHg		77 (10)	78 (10)	77 (10)	0.039
HbA1c (SD), %		7.10 (1.31)	5.93 (0.39)	5.42 (0.37)	<0.001
Casual PG (SD), mg/dl		156.0 (57.9)	115.5 (22.4)	99.9 (16.5)	<0.001
Fasting PG (SD), mg/dl (n = 1,249)		139.8 (32.4)	106.0 (12.3)	93.1 (8.0)	<0.001
FH of diabetes, n (%)		162 (39.1)	94 (28.1)	1,030 (14.8)	<0.001
Walking time, n (%)					
<0.5 hrs		63 (15.2)	47 (14.1)	943 (13.5)	
0.5-0.9 hrs		104 (25.1)	85 (25.5)	1,517 (21.8)	
1.0-1.9 hrs		83 (20.1)	80 (24.0)	1,549 (22.2)	
≥2.0 hrs		164 (39.6)	122 (36.5)	2,957 (42.5)	
Parity status					
Parous women without a history of having a macrosomic infant		347 (83.8)	289 (86.5)	6,117 (87.8)	
Nulliparous women		29 (7.0)	22 (6.6)	467 (6.7)	
Parous women **with** a history of having a macrosomic infant		38 (9.2)	23 (6.9)	382 (5.5)	

BP: blood pressure, FH: family history, PG: plasma glucose

## Discussion

 The present study investigated the relationship between a history of having a macrosomic infant and the risk of diabetes. First, we described the prevalence of women with a history of having a macrosomic infant in the 5-year survey of the JPHC Diabetes cohort, with the prevalence being 6.0 %. Since there has been no study which reported this type of descriptive epidemiology, it might be difficult to validate our estimate. According to the vital statistics of Japan in 1970 [8], when the cohort participants were in their reproductive age, 3.04% of newborns had birth weight ≥ 4000g. The total fertility rate of Japan in 1970 was 2.13 [9]. If it is assumed that each delivery was independent (of course, the reality of each delivery is not always independent), the frequency of parous women with a history of having a macrosomic infant was 6.4%, which is calculated by the following formula: 1-(1-0.0304)^∧2.13^. This figure appears to be close to the estimate we counted from the JPHC Diabetes cohort. 

With regard to the relationship between a history of having a macrosomic infant and the risk of diabetes, our cross-sectional analysis reported that parous women with a positive history were 1.4 times more likely to have diabetes compared with women without. On the other hand, the longitudinal analysis showed that parous women with a positive history had a modest but non-significant increased risk of developing diabetes with an OR of 1.2. The discrepancy in the results between the cross-sectional analysis and the longitudinal analysis could be explained by the following reasons. First, women who delivered a macrosomic infant may develop diabetes early after the delivery. A meta-analysis [10] on the relationship between gestational diabetes and the incidence of diabetes demonstrated that the progression to diabetes increased steeply within the 5 years after delivery and reached a plateau, suggesting that unmasked β-cell dysfunction during pregnancy appeared to progress in the early years of their postnatal life. In the present study, the average age of the study participants in the longitudinal analysis was 61.6 years. Therefore, women with a history of having a macrosomic infant, who might have latent β-cell dysfunction, had already developed diabetes before the start of the present study. This was supported by the result shown in Table 6. Women who had diabetes at baseline and were excluded from the longitudinal analysis had a high percentage of having a positive history, suggesting that women with a positive history and at high-risk for diabetes had already developed diabetes at baseline. This might lead to a non-significant result in the longitudinal analysis. Another reason could be a lack of statistical power. The longitudinal analysis, which counted a new case of incident diabetes, could not have collected enough cases to detect a significant difference in the incidence of diabetes between women with a positive history and those without. As shown in Table 7, the statistical power to detect the effect size of 1.2 in the longitudinal analysis was only 11.6%. Therefore, the non-significant relationship can be interpreted as a lack of statistical power to detect the modest relationship rather than a null result. In general, the result of a longitudinal observational study is more valued than that of a cross-sectional analysis. However, considering these reasons, it might not be the case in the present study.

**Table 7:** Statistical power of the longitudinal analysis to detect a difference.

Effect size to detect		Power
Odds ratio		%
1.1		5.3
1.2		11.6
1.3		21.0
1.4		32.8
1.5		45.7

 As for nulliparous women, the present study did not show a significant difference in the risk of diabetes compared with parous women without. Among the nulliparous women, there seems to be a certain proportion of women who would have had a macrosomic infant if they had experienced delivery. Therefore, the risk estimate of nulliparous women for diabetes was expected to be between that of parous women with a positive history and that of parous women without. However, the increase in the risk observed in the present study was close to null probably because the proportion of the women who would had a macrosomic infant if they had experienced delivery was too small to show a significant increase in the risk.

 The present study had several limitations. First, a history of having a macrosomic infant was not evaluated by medical records but a self-reported questionnaire, which could involve recall bias. In general, it is likely that a woman who experienced having a macrosomic infant might forget the fact and not report it. On the other hand, it is unlikely that a woman who did not have a macrosomic infant wrongly reports that she did. This non-random misclassification could lead to underestimation of the result in the present study. In addition, random misclassification brings the result toward the null. Considering these points, the risk estimates obtained from the present study could be underestimated. Second, information on life-style behaviors such as alcohol consumption or smoking status during pregnancy was not available in the present study. These factors may bring our risk estimates in an unpredictable direction. Alcohol consumption above a certain amount is a risk factor of diabetes [11]. Drinking alcohol during pregnancy is associated with the risk of a low birth weight [12]. Therefore, alcohol consumption may have weakened the apparent relationship. Adjustment for alcohol consumption might result in an increase in the risk estimate. On the other hand, eating behavior related to alcohol consumption could bias the estimates into the opposite direction. Furthermore, unidentified confounders may exist in the relationship between a history of having a macrosomic infant and the risk of diabetes. Third, the number of events was relatively small in the longitudinal analysis, which might affected the validity of logistic regression models [13]{Peduzzi, 1996 #28}.

 Despite these limitations, there were strong points in the present study. The case ascertainment of diabetes was performed by a standardized laboratory method in combination with a questionnaire. Using a large sample from the general population suggests that the results in the present study could have an external validity. Moreover, this is the first study to examine the relationship between a history of having a macrosomic infant and the risk of diabetes in a population-based cohort.

In conclusion, we investigated the relationship between a history of having a macrosomic infant and the risk of diabetes. In the cross-sectional analysis, women with a positive history were 1.4 times more likely to have diabetes. The longitudinal analysis showed a modest but non-significant increased risk of developing diabetes. The non-significant result might not necessarily indicate a null result. Further research to quantify the accurate risk estimates is required. Finally, not only women with a history of having a macrosomic infant but also medical professionals who see these women should keep in mind the possibility of an increased risk of diabetes.
